# Digging in or building bridges? A scoping review of thematic analysis

**DOI:** 10.3389/frma.2025.1617380

**Published:** 2025-11-20

**Authors:** Christian Herzog, Christian Handke, Erik Hitters

**Affiliations:** 1 University of Münster, Münster, Germany; 2 Erasmus School of History, Culture and Communication, Erasmus University Rotterdam, Rotterdam, Netherlands

**Keywords:** qualitative methods, data analysis, thematic analysis, scoping review, bibliometrics, mixed methods, natural language processing, content analysis

## Abstract

This scoping review deals with a major trend in qualitative data analysis: thematic analysis (TA) that provides a general framework to develop relatively transparent processes; TA thus helps mitigate long-standing concerns with allegedly subjective aspects of qualitative research. The review examines articles published in the top-ranked academic journals in the research area “communication” (*n* = 342). It illustrates that TA has quickly become more popular over recent years, complementing longer established qualitative methods. The analysis also reveals that TA is a flexible tool that has been successfully applied to make sense of a wide array of qualitative data. Building on these findings, we deduce practical advice for researchers applying TA and make suggestions on how to improve on current TA practices by, first, documenting common features of successful TA articles (best practice), and, second, identifying apparent superficialities and untapped potentials.

## Introduction

Thematic analysis (TA) is an increasingly popular method for the identification and interpretation of patterns of meaning in qualitative data. There have been various competing uses of the term, some rooted in a quantitative paradigm (e.g., Boyatzis, 1998; Fugard and Potts, 2015).^1^ However, as we will document in the following, Braun and Clarke's (2006) TA approach (cf. Braun and Clarke, 2019, 2023b), which emphasizes a qualitative methodology, has been so influential that it is virtually synonymous with TA by now. It involves an iterative engagement with the data across six phases: (1) familiarization with the data, (2) initial coding, (3) searching for themes, (4) reviewing potential themes, (5) defining and naming themes, and (6) producing the report (Braun and Clarke, 2006).

A number of literature reviews have addressed the use of various qualitative data analysis methods in communication research and related fields (e.g., Bradbury-Jones et al., 2017; Jensen et al., 2013; Ngenye and Kreps, 2020; Snelson, 2016). However, we have found only two scoping reviews focusing on TA. First, Bowman et al. (2023) investigated 78 papers published in the Association of Computing Machinery (ACM) CHI conference proceedings on Human Factors in Computing Systems. The authors report on limited documentation of methods and a lack of explicit reflection on methodology (reflexivity) in healthcare human-computer interaction. Second, Braun and Clarke (2023a) reviewed 100 TA papers in five journals from health psychology. The authors lament the predominance of qualitative-positivist research methodologies in this field. In this context, they emphasize TA's potential to promote methodological diversity but worry that in TA practice, “positivism invidiously and invisibly directs research practice” (Braun and Clarke, 2023a, p. 698). They identify areas of problematic practice in TA applications within health psychology, call for greater “qualitative research sensibility” (Braun and Clarke, 2023a, p. 701; see also Braun and Clarke, 2013) and develop a rich set of recommendations for authors, reviewers, and editors to apply adequate and unique quality criteria for TA.

This scoping review documents TA application in a different field and adopts a different perspective. We focus on articles in the research area of media and communication studies, which—particularly in the area of reception studies—is more specialized on qualitative data analysis (Jensen, 2021) than for instance health psychology. Here, TA does not serve to promote qualitative research in predominantly quantitative research areas, but it also complements and possibly replaces other methods of engaging with qualitative data. Thus, deliberate or heedless imposition of positivist-quantitative methodology should be less of a concern. Instead, TA arguably has the potential to bridge traded schisms between different empirical methodologies.

In its current state, TA is in a fascinating position as it strikes pragmatic and constructive compromises between empirical research paradigms that have often been presented as antagonistic and mutually exclusive. On the one hand, TA results are qualitative: compared to quantitative content analysis, for instance, TA preserves and reports relatively much detail and nuance in qualitative data that it categorizes into themes.^2^ Fully developed themes, the final outcome of the analysis, are supported by relevant quotes from the data (Connelly and Peltzer, 2016; Mishra and Dey, 2022). On the other hand, compared to longer established methods of manual qualitative data analysis—such as qualitative content analysis (Vaismoradi et al., 2013), discourse analysis, narrative analysis, or grounded theory—TA puts greater emphasis on systematically documented proceedings (cf. Nowell et al., 2017) akin to quantitative research traditions, and it has succeeded in popularizing a relatively detailed, universally applicable protocol for conducting and reporting on qualitative data analysis.^3^ Arguably, TA therefore mitigates a common concern regarding manual qualitative data analysis, as it guides researchers to reflect on choices and to discuss them in a reasonably transparent manner (Gough and Madill, 2012), facilitating constructive criticism by others. With the proliferation of unstructured, qualitative data in the context of digitalization (cf. Crowston et al., 2012), there is thus great scope for further TA applications and cross-fertilization with automated natural language processing (NLP), including the use of learning algorithms. To bring any of this to fruition, however, TA applications must convincingly document the data under study and their analysis (transparency) and inform readers on the ifs and buts of the authors' choices and priorities.

With these challenges in mind, using the Preferred Reporting Items for Systematic reviews and Meta-Analyses extension for Scoping Reviews (PRISMA-ScR) checklist (Tricco et al., 2018) as a guiding principle, we systematically review a “large, complex [and] heterogeneous” body of literature (Peters et al., 2015, p. 141), in order to illustrate the “volume, nature and characteristics” (Arksey and O'Malley, 2005, p. 30) of TA scholarship in top-ranked communication journals. By focusing on the “top-end” of prestigious journal articles, we get cues on “best practice” in TA applications and develop hands-on advice for researchers seeking to develop a state-of-the-art analyses of qualitative data in communication research. However, as Braun and Clarke (2019) have worried, publication success of TA applications has not reliably coincided with good practice according to their general quality criteria. Following our analysis, we then discuss apparent shortfalls and untapped potential even within the top-end of TA applications according to publication success. Our specific research questions are the following:

1. How much is TA applied?
2. For what purposes and how do successful communication articles apply TA (and how can this help us explain the growing appeal of TA)?
3. Looking forward, what hands-on advice can we deduce for researchers considering TA applications in two respects:
  a. Regarding common features in impactful TA articles conceived as “best practice,”
  b. Regarding apparent limitations in TA applications, in particular in terms of transparency and reflexivity?

To be sure, the two aspects of research question 3 entail a shift in perspective on what constitutes “quality” in TA applications. We first elicit lessons to be learned from quality judgements by other parties—journal editors and reviewers. We then also compare the articles that have made it into prestigious communications journals (and thus into our data set) against general quality criteria associated with TA. Arguably, both perspectives will be useful to inform researchers, editors and educators engaging with qualitative data analysis.

The paper proceeds as follows. First, we document the construction of the data set. Second, we map the number TA articles over time, per number of authors, per country, and per topical areas. Third, we report the primary and secondary data sources used in TA articles. Fourth, we identify and discuss the most cited references within the data set. Fifth, we address key features of the most cited articles within the data set. Sixth, we deduce practical advice for scholars considering TA applications. Finally, we take stock of major challenges and desirable areas of advances in TA methods.

## Construction of the data set

**Figure 1:**
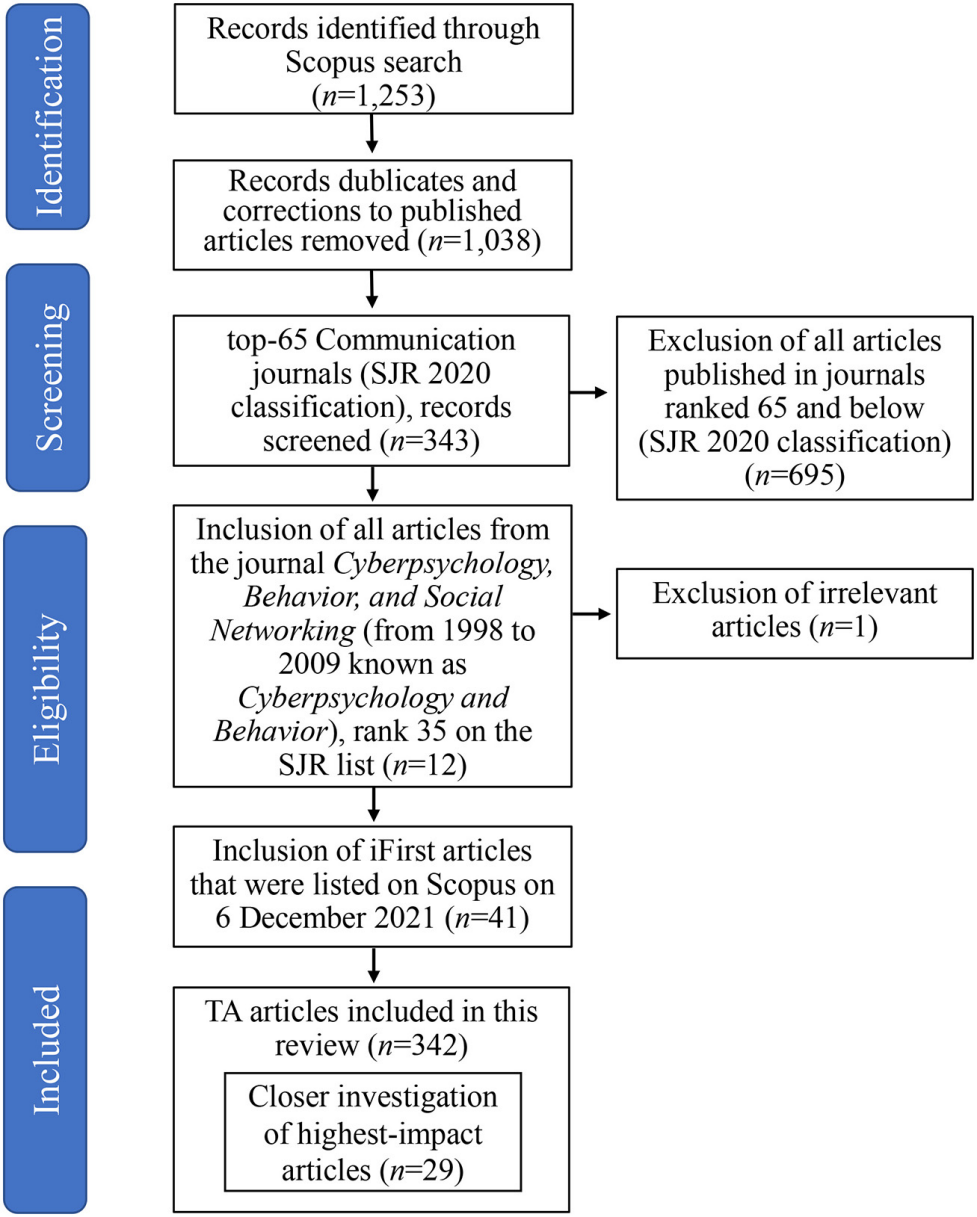
PRISMA flow chart.

There is only a limited number of international scientific databases which allow to obtain robust and reliable bibliographic data for a study of this kind, with Scopus and Web of Science being the major databases in the social sciences, including the field of communication (Singh et al., 2021; Zupic and Cater, 2015). As various authors have noted that Scopus provides more comprehensive and higher quality coverage (Falagas et al., 2008; Mongeon and Paul-Hus, 2016), in this study we exclusively used Scopus to identify relevant academic articles to include in the data set. We used Boolean searches on Scopus and searched for journal articles in the research field of Communication (Scopus code 3315), which feature the expressions “thematic analysis” or “thematic analyses.” The final search was conducted on 6 December 2021. The exact search query used in the advanced search interface on Scopus is “( ( TITLE-ABS-KEY (“thematic analysis” OR “thematic analyses”) ) AND ( PUBYEAR < 2024 ) ) AND ( SUBJTERMS ( 3315 ) ) AND ( LIMIT-TO ( DOCTYPE, “ar”) ).” As indicated in the PRISMA flow chart displayed in Figure 1, after deleting duplicates and removing corrections to published articles, this search yielded 1,038 distinct journal articles.

Scopus makes available much meta-data on each article. However, in order to develop a thorough analysis of key features, we also needed to construct some additional variables by human coding of full text articles. Furthermore, for our purpose of identifying successful practices in TA work, publications in more prestigious journals are of greatest interest. To limit the labor required with minimal adverse effects on the quality of the data set, we excluded articles from less prestigious journals and limited the scope of investigation to the top-65 communication journals according to the 2020 classification of the Scimago Journal and Country Rank (SJR) (Guerrero-Bote and Moya-Anegón, 2012).^4^ Focusing on top-ranked journals and top quality papers only is a common strategy to restrict the size of the data based on journals particularly when a study reports findings of articles and the search on Scopus produces a number of articles that is difficulty to handle.^5^

The data set features 41 iFirst articles that, on 6 December 2021, had not been published in a particular journal volume and issue yet but were listed on Scopus. Hence, the data set includes articles with the final publication years 2022 and 2023. Applying judgement sampling (Maul, 2018), we excluded one article published in 1984 (McAdams and Losoff, 1984) as the article does not feature TA in the contemporary meaning of the term. After these curation steps, the final data set consists of 342 prestigious TA articles, which are classified by Scopus as articles (330) or as reviews (12). We confirmed by reading the abstracts that the 12 reviews included are broader literature review articles with original content, rather than book or policy reviews. All articles were published in English.^6^

## Bibliometric features of the data set

**Figure 2:**
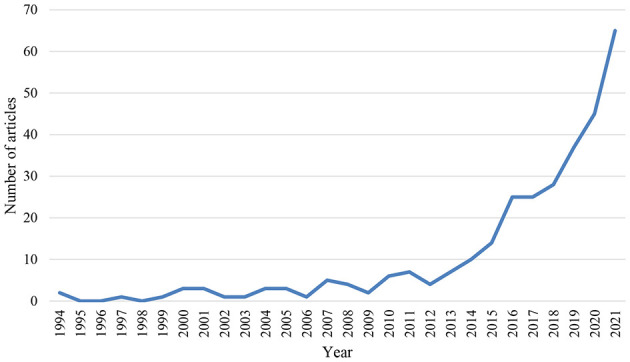
Number of TA articles in communication journals per year. Figure 2 only displays full years. Not displayed are the number of [iFirst] articles for 2022 (34) and 2023 (5).

In the data set under study, the number of relevant articles per year strongly increased over time (see Figure 2). Since 2013 there has been a rather consistent, approximately exponential growth pattern.

The 342 articles in the data set were published in 46 different journals (see Table 1 and Supplementary material A). Journals that feature most TA articles are *Health Communication* (55 articles), *Journal of Social and Personal Relationships* (27 articles), and *New Media and Society* (23 articles). Among the top-65 communication journals, 19 journals did not feature any articles that fulfilled the search criteria.

**Table 1:** Descriptive statistics of key variables regarding journals and articles in the data set.

	**Score**		**Mean**	**Min**	**Max**
**Journals**					
Number of journals	• 65 top-ranked journals in Scopus • 46 journals with relevant articles		-	-	-
Number of articles	342	All 65 Journals	5.25	0	55
		Journals with relevant articles	7.43	1	55
Citations	6,888		149.74	2^b^	665^b^
Article age^a^	-		-	1.5^b^	25^b^
**Articles**					
Citations	6,888		20.14	0	459
Article age^a^			6.76	1^c^	30
Citations per article and year			2.74	0	39.80
**Authors**					
Total different authors	873	Authors per article	2.66	1	14
Citations per author(s)	-		7.89	0	459

^a^Journal means calculated as 2024 minus article publication year(s); also see note ^c^. ^b^Journal means. ^c^The data set features iFirst articles that were listed in Scopus in 2022 but finally published in 2023.

TA research is often carried out by small teams (see Tables 1, 2). Overall, the data set features 873 different authors. The mean number of authors per article is 2.66. In the data set, 79 articles were single-authored and 125 had two authors, jointly accounting for 59.65% of all articles. The number of individual authors featuring only once is 843. Thus, there is little indication that individual authors would specialize on TA. What is more, there is no apparent positive effect of author team size on impact as indicated by citation counts (see Table 2). To the contrary, single authored articles are on average cited more frequently (mean 30.82) than multi-authored papers (mean 16.93), and this pattern also holds controlling for article age.

**Table 2:** Number of authors per article and its association with citation scores.

**Number of authors**	**Article count**	**Total number of “authorships”^a^**	**Citations**				**Article citations per year since publication**		
			**Total**	**Mean**	**Min**	**Max**	**Mean**	**Min**	**Max**
1	79	79	2,435	30.82	0	459	3.41	0	39.80
2	125	250	2,737	21.90	0	201	2.69	0	15.29
3	61	183	698	11.44	0	133	2.34	0	26.60
4	37	148	439	11.87	0	66	2.31	0	12.00
5	21	105	368	17.52	0	54	3.31	0	18.00
6	8	48	120	11.6	1	36	2.21	0.33	4.00
7 or more	11	100	91	8.27	1	25	1.69	0.25	3.13
Total	342	911	6,888	20.14	0	459	2.74	0	39.80

^a^The same author may be associated with multiple articles, so that there are fewer authors than “authorships.”

The majority of authors reside in the United States (165 articles), followed by the United Kingdom (65 articles) and Australia (24 articles) (see Table 3). Authors from BRICS countries, such as China, play a much smaller role here than in academic publications overall. Perhaps surprisingly, some large European countries are missing from the top ten countries of origin list. In the case of Germany, this absence can be explained by the widespread use of “qualitative content analysis” in that country, an alternative to TA as featured in Mayring (2010).

**Table 3:** Top ten countries by residence of authors.

**Rank**	**Country^a^**	**Articles**	**Citations**
1	United States	165	2,867
2	United Kingdom	65	1,719
3	Australia	24	359
4	Canada	15	253
5	New Zealand	14	253
6	Israel	11	105
7	China	7	37
8	Czech Republic	7	36
9	Finland	7	239
10	Netherlands	6	51

^a^refers to all authors. Geographical locations were mapped by means of VOSviewer.

### Topical areas

To illustrate the suitability of TA to address various topical areas, we used the International Communication Association's (n.d.) system of classifying communication research topics (see Supplementary material B).^7^ Out of 25 well-established topic “divisions” and eight emerging “interest groups,” only three divisions and one interest group are not represented in our data set. Apparently, TA is widely applicable across virtually all topical areas.

Another interesting pattern is that some of the divisions and interest groups with the least TA articles in our data set are on digital ICT (the divisions Computational Methods with two articles, Information Systems with zero, and the interest group Human-Machine Communication with one article). We argue below that there is great potential for complementarity and cross-fertilization between TA and ICT-based NLP. This potential remains largely untapped.

### Data sources

Having identified the key topical areas in applied TA research published in top-ranked communication journals, in this section we investigate the data sources of the articles under study. The data set includes three non-empirical theoretical-conceptual articles,^8^ and six articles which, in our reading, do not present reasonably comprehensive information on data.^9^ Excluding these articles, we have manually classified the remaining 333 articles according to two categorizations. First, we have distinguished articles that analyze primary data or secondary data. Whereas primary data is generated by researchers first-hand, secondary data has previously been gathered by other parties (see Rabianski, 2003). Second, we classified articles according to data collection methods (see Table 4). In the following, we separately report the frequency of data collection methods for primary and secondary data.

**Table 4:** Primary and secondary data sources.

**Data sources**		**Articles analyzing the data source (Percentage share)^a^**	**Articles analyzing the data source and (an)other data source(s) (Percentage share)^a^**
Primary data sources	Interviews	144 (41.11)	26 (7.60)
	Focus groups	39 (11.40)	17 (4.97)
	Surveys/ Questionnaires	38 (11.11)	6 (1.75)
	Writing tasks	11 (3.22)	5 (1.46)
	Observations/ Field notes	11 (3.22)	6 (1.75)
	Other	5 (1.46)	n.a.
Secondary data sources	Social media posts/ Comments	66 (19.30)	19 (5.56)
	Press coverage	54 (15.79)	30 (8.82)
	TV/Radio/ Video	13 (3.80)	7 (2.05)
	Academic articles	10 (2.92)	4 (1.17)
	Gray literature/ Policy documents	7 (2.05)	5 (1.46)
	Websites	6 (1.75)	6 (1.75)
	Other	13 (3.80)	n.a.

^a^Percentage share refers to the full data set (*n* = 342).

#### Primary data

TA has been employed for a wide variety of primary data analyses. The most frequent primary data collection method are interviews, which feature in a total of 144 articles (42.11% of all 342 articles). Many variations of interview techniques are present, and virtually all articles fulfill the basic criteria of methodological transparency (Gubrium et al., 2012): they specify sample criteria, the duration of interviews, and how the interviews were conducted. The number of interviewees varies between 3 and 114.^10^ 12 articles use data generated in no more than 10 primary interviews, four use data from 100 interviews or more. The average number of interviewees per articles is 30.^11^

Whereas 118 articles solely analyze interviews, in 26 articles interview data is combined with one or more other data sources. In 10 articles interviews are combined with focus groups. From these 10 articles nine use only interviews and focus groups as data sources and one article additionally relies on survey data. In total, three articles combine interviews with survey data. Four articles analyze news articles as additional data source (one of these in combination with policy documents) and three articles published in *Health Communication* combine interviews with observational data. The remaining articles combine interviews either with research diaries, experiments, writing tasks, consultations, webposts, or Facebook comments. In one exceptional case, Doan and Toledano (2018) analyze a civic crowdfunding campaign, the authors include interviews, Facebook messages, Facebook comments, written and audiovisual materials, website contents and comments on Givealittle.co.nz, news articles and blog posts in their data set.

Focus groups and surveys/questionnaires are also relatively common methods of primary data collection, respectively occurring 39 and 38 times. Writing tasks and observations/field notes each occur 11 times. “Writing tasks” provided to informants encompass six articles analyzing (reflective) written accounts/narratives, four research diaries, and one story-completion task (the latter in Eyal and Ben-Ami, 2017). In eight of these studies all research participants have been students, and there are often links to education and pedagogy. “Observations/field notes” refers to qualitative data produced by researcher(s) themselves. Seven of these articles draw on notes from observational sessions and interventions (five thereof in clinical settings), two articles report ethnographic case studies, and another two analyze video (streaming).

Regarding all articles, which analyze primary data sources, 24.19% articles use more than one type of data source. Notably, within a single article, analyses of focus groups, “observations/field notes” and “writing tasks” are relatively often combined with analyses of other types of data. Several focus group studies, for instance, also analyzed data from interviews (*n* = 10), surveys/questionnaires (*n* = 4), writing tasks (*n* = 2), observations/field notes (*n* = 2), and press coverage (*n* = 1). An interesting “outlier” in terms of an ambitious combination of multiple sources of primary data is Yang and Yeh's (2021) study of intercultural communication skills of English language students, which relies on reflective essays, oral presentations, concept maps, and YouTube videos produced by the research participants as data. The latter two data sources fall in the category “other,” which also includes two studies that use email exchanges with research participants as data.

#### Secondary data

A total of 54 articles rely on press coverage as a source of data (15.79% per cent of all 342 articles). Articles which analyze press coverage can be distinguished into studies that investigate news articles published in print and online media, including editorials and opinion pieces (41 articles), magazine articles (seven articles), and (journalism) blog articles (six articles). The average number of press items analyzed is 176, ranging from 30 (see Jha, 2007) to 621 (see Mejias and Banaji, 2019).

“Social media posts/comments” analyzed by articles in the data set derive from six platforms: X (formerly Twitter) (23 articles), Facebook (nine articles), Reddit (five articles), Youtube (four articles), Instagram (three articles), and Weibo (two articles). Of these 66 articles in total, 17 articles analyze posts and comments in online discussion forums (including discussions on news websites, blogs, and forum discussion threads), and three articles analyze comments posted in response to online press articles. Furthermore, several articles employ TA to make sense of “tv/radio/video” content, of academic articles (for conducting systematic literature reviews), of “gray literature/policy documents,” and of websites. The category “other” includes textbooks (three articles), press releases (three articles), WhatsApp messages, political advertisements, and convention presentations (one article each). In terms of data which includes visual components, the articles by Kristensen and Mortensen (2021) and Murthy et al. (2020) analyze memes and emojis, while Gavin et al. (2019) investigate online dating profiles.

Combinations of various types of *secondary* data are relatively common. 42.01% articles that analyze secondary data jointly analyze at least two data sources (see Table 4). For instance, of the 54 articles which analyze press coverage, 30 also analyze other secondary data, including “social media posts/comments” (six articles), academic articles (five articles), interviews, “gray literature/policy documents,” “tv/radio/video” transcripts (four articles each), websites (three articles) as well as focus groups, and press releases (one article each). The secondary data referred to above almost entirely derives from spontaneously generated verbal expressions captured as media content (created without prompting by researchers). In our data set, we find no apparent reuse of qualitative data collected by other (academic) researchers.

### Most cited references and what they tell us about TA methods

The 342 articles in our data set in total feature 16,067 references. We deduplicated these references with a thesaurus file in VosViewer.^12^ Table 5 displays the most cited references in the data set. Eight of the most cited references refer to publications about research methodology.^13^ A striking observation is that Braun and Clarke's (2006) article “Using thematic analysis in psychology” published in *Qualitative Research in Psychology* is by far the most cited source^14^ with a total of 121 citations and over 7.12 citations per year since publication—almost as many as all other sources featured in Table 5 combined (142 total citations and 8.22 citations per year). Lincoln and Guba's (1985) broad methodology book ranks in second place by total citations (21). Boyatzis (1998) coding manual for qualitative data analysis, which features TA in the very title and is rooted in a quantitative paradigm, has been referenced by 16 articles. Two further, general research method books are among the list of the most cited publications in the data set: Patton (1990) (11 citations) and Lindlof and Taylor (2011) (8 citations).^15^

**Table 5:** The most frequently referenced publications in the data set.

**Rank**	**Reference**	**Citations**	**Citations per year**
1	Braun V., Clarke V. (2006). Using thematic analysis in psychology. *Qualitative Research in Psychology*, 3(2), 77–101.	121	7.12
2	Lincoln Y. S., Guba E. G. (1985). *Naturalistic inquiry*. Sage.	21	0.55
3	Boyatzis R. (1998). *Transforming qualitative information: Thematic analysis and code development*. Sage.	16	0.64
4	Corbin J., Strauss A. (2008). *Basics of qualitative research: Techniques and procedures for developing grounded theory* (3^rd^ ed.). Sage.	15	1.00
	Owen W. F. (1984). Interpretive themes in relational communication. *Quarterly Journal of Speech*, 70(3), 274–287.	15	0.38
5	Patton M. Q. (1990). *Qualitative evaluation and research methods* (2^nd^ ed.). Sage.	11	0.33
6	Charmaz K. (2014). *Constructing grounded theory* (2^nd^ ed.). Sage.	10	1.11
	Glaser B., Strauss A. (2017). *The discovery of grounded theory*. Aldine.	10	1.67
7	Lindlof T. R., Taylor B. C. (2011). *Qualitative communication research methods* (3^rd^ ed.). Sage.	8	0.67
8	Goffman E. (1959). *The presentation of self in everyday life*. Doubleday.	7	0.11
	Goldsmith D. J. (2004). *Communicating social support*. Cambridge University Press.	7	0.37

Three of the most cited method publications are classic grounded theory textbooks: Corbin and Strauss (2008), Charmaz (2014), and Glaser and Strauss (2017)^16^. This underscores the importance of grounded theory as “the other” methodological foundation of many research applications of TA within our data set. TA and grounded theory relate to each other in a mixture of complementarity and rivalry. On the one hand, they are both suitable for open coding within an inductive analytical strategy and may complement each other. On the other, TA may have come to rival and partially substitute the longer established grounded theory-framework(s) for engaging with qualitative data. In a nutshell, there are two main distinctions. First, TA as in Braun and Clarke (2006), advocates a more general, systematic and almost formulaic approach to qualitative data analysis than much of the grounded theory literature. Second, Braun and Clarke (2006) seem agnostic as to the use of theory, whereas grounded theory is characterized by inductive use of theory. In the next section, we will show that pure inductive use of theory hardly occurs in high-impact TA articles.

### Key features of the sub-set of most cited articles within the data set

This section takes a closer look at the most cited articles within the data set, for which we conducted a summarizing full-text content analysis. To get indications of best practice^17^ in the TA publications under study while aiming for a manageable workload, we drew a sub-sample of all top-20 articles in our data set according to (a) total citations and (b) citations per year since publication (see Supplementary material C). Due to overlap of articles between these two rankings, this results in 29 highest-impact papers, which account for 8.48% of articles in our full data set but for 44.70% of total citations.

#### Use of theory

Braun and Clarke (2013) emphasize the potential for inductive development of themes (that can lead to novel theoretical categorizations) in TA applications. Nonetheless, proper inductive research strategies are clearly in the minority among the high impact articles. All articles discussed related, prior empirical work, and the majority of articles referred to theory before reporting on empirical work (see Supplementary material D). This may partly be an artifact of the perceived expectations of editors and reviewers for articles to adhere to a conventional structure familiar from deductive (and quantitative-statistical) research.

However, the use of theory in TA articles considerably deviates from research in traditional (social) sciences. No article explicitly developed any testable propositions or even sought falsification of theory. The typical approach followed in the high impact articles is to invoke theory as a guiding framework for subsequent TA data analysis. Often, various rival “theoretical perspectives” on the topic at hand are invoked, with the authors “taking sides” at the outset. Data analysis results are then presented as verification of theory or documentation that favored theory works to foster understanding. A typical example is provided by Kassing (2002, p. 202): “These findings provide support for the exit-voice-loyalty model (and subsequent revisions) of employee dissatisfaction (Farrell, 1983; Graham and Keeley, 1992; Hirschman, 1970).”

Thus, the high-impact TA articles neither adhere to conventional, deductive use of theory nor to the traditional rationale of qualitative research that “the primary purpose […] is discovery, not hypothesis testing […,] not trying to control variables, but to discover them” (Corbin and Strauss, 2008, p. 317, 318). This methodological flexibility entails considerable challenges. In most of the 29 articles, it remains unclear to what extent theories have been identified *after* data analysis had commenced. Even in articles, where authors explicitly refer to inductive research strategies, theory is presented in sections preceding the original empirical work. Apparently, that is a common practice, but it seems to obfuscate the actual research process. It would be more transparent to explicitly document the extent of inductive, iterative, or deductive elements in the research process (Shen et al., 2024). In any case, the most-cited TA articles invoke rather diverse theories, indicating the flexibility of TA.

#### Flexible application across multiple topics and on broad ranges of data

The high impact articles also document that TA is suitable for addressing a broad array of topics and types of qualitative data. The vast majority of highly cited articles (27 of 29) apply TA for original empirical work. The remaining two articles are literature reviews (Pavlenko, 2007; Tandon et al., 2021). Various methods of data collection feature among these high-impact papers. Of the 27 articles that present original TA applications, nine used data generated in interviews conducted or supervised by the researchers, seven collected data by manual or automated “web scraping,” six used data produced by surveys (where authors were not always involved in data collection), three mostly analyzed academic literature, two investigated other types of published written-text documents, and one analyzed broadcasts.

Regarding the count of separate units of analyses covered, even though there is considerable variance, articles analyzing interview data tend to have low scores. Data from web scraping tends to be associated with much higher scores, and surveys tend to fall between these two extremes. Overall, this illustrates the flexibility of TA as means to analyze many different types of data sets with varying sizes and units of analysis.

#### Lack of reflexivity

Most authors apparently consider the concept of TA as rather self-explanatory. In 13 articles, we have not found any reference to other publications that provide general discussions and guidelines for TA. There are virtually no detailed, reflexive discussions on TA and its specific applications. The main exceptions are the two articles focusing on general aspects of TA and related data analysis methods while not employing TA themselves (Pavlenko, 2007; Tandon et al., 2021). Six articles refer to Boyatzis, five to Braun and/or Clarke, and four to Pavlenko in the respective lists of references. The list of reference by Coulson and Knibb (2007) is the only additional paper that features references to two of these influential (teams of) authors (Braun and Clarke, as well as Boyatzis). Overall, minute discussions of various perspectives on TA are hardly deemed necessary by authors of highly-cited articles employing the method.

#### Mixed methods

TA is occasionally combined with other methods of analyzing qualitative data in a mixed-method design. Among the 27 high-impact articles featuring original TA applications, five combined TA with quantitative content analysis. Beyond basic counts of themes' appearances, other complementary data analysis methods, like descriptive- or inferential statistics, only feature in one article each—LeFebvre (2018) and Timmermans et al. (2021) (see Supplementary material C). These are low numbers, considering that successful TA results in countable categories and that until recently, the term TA has occasionally also been used to refer to quantitative content analysis.

There are some probable reasons besides the methodological preferences and specializations of authors. First, TA may simply take up all of the feasible word count for articles (Braun and Clarke, 2023a). Second, typical TA data sets may often be too small to require or enable additional quantitative data analysis.

## Discussion and suggestions for successful TA practices

Our investigation yields several useful insights and actionable recommendations for researchers engaging with TA. On the one hand, based on common features of the successful TA articles in our data set, we can infer on best practices so far. On the other hand, we discuss apparent shortcomings. Most articles in our data set deviate from the transparency and reflexivity ideals of TA. Most articles in our data set deviate from the transparency and reflexivity ideals of TA (see Herzog et al., 2019; Herzog and Kelly, 2023). In the following we discuss on how to improve current TA practices.

### Going with the flow

TA is an increasingly applied method to analyze qualitative data by human reading. It is a flexible method and has been successfully applied for a great variety of research topics and types of qualitative data sets. TA has served well for authors emphasizing deductive engagements with theory, for inductive theorizing, as well as iterative research processes. As we document in this article, TA applications have required little justification and explanation to become acceptable to journal editors and reviewers.

### No methodological specialization required

According to our analysis, TA does not require much specialized skills, substantiating common but casual assessment in the literature (e.g., Braun and Clarke, 2013; Castleberry and Nolen, 2018).^18^ TA has been successfully applied in quickly growing number of articles, most of which have been generated by small teams of researchers. Furthermore, many authors have successfully applied TA only once for a specific paper, hence it appears reasonably to conclude, that no specialization is required.

### TA as a means to promote the transparency of qualitative data analysis and enable reflexivity

A key reason for the increasing appeal of TA is that it promotes the systematization of manual qualitative data analysis. TA has the potential to foster transparency of data analysis processes and thus to enable reflexivity as well as constructive criticism by others. To harness these advantages, we recommend to specifically address transparency and reflexivity in the methodological justifications, and to anchor this in the relevant literature.

### Appropriate outlets

An obvious question for any academic considering TA is, which publication outlets to target. The answer will depend on many considerations. Nonetheless, this scoping review entails some insights on the propensity of various communications journals to accept TA applications, which seems to vary considerably. In the data set under study, 13 journals have published at least 10 TA articles, which seems to suggest some appreciation by the associated editors and reviewers, while 19 journals have not yet published articles prominently featuring that method.

### Specifying the use of theory

One area, where there is great potential for improving current TA practice, is the handling of theory. Most articles in our data set are rather obscure on whether they made mostly inductive, iterative, or deductive use of theory. In contrast to, for example, grounded theory, TA makes no specifications on the use, integration or even development of theory. That makes it even more important for authors to document this essential aspect of the research process. Readers should provide some information on how preconceived ideas from theory guided the research process, and how authors deliberately replace or extend upon extant theory.

### Reflexivity in data analysis

In its current state TA makes specification for all stages throughout the analytical process, from data gathering to writing up. Within any of these stages, there are multiple options. For instance, regarding the coding stage: reliability coding involving multiple coders, codebook TA, or reflexive coding (Braun and Clarke, 2024). Authors ought to discuss what options they considered and what pragmatic or methodological considerations affected the priorities set. In the vast majority of article in our data set, this information remains rudimentary.

On the one hand, greater reflexivity is required to develop a good match between specific TA applications and the needs and intentions of researchers—and to enable readers to assess the credibility and limitations of results. On the other hand, explicit discussions of choices and any lessons learned during the research process are required to promote further development of TA.

### Explore complementarities and mix methods

While TA facilitates cross-disciplinary and multi-method research, apart from the common methodological basis with grounded theory literature (see Table 5), linkages to other methods or methodologies are rarely explored in our data set. Within qualitative research, more explicit debates are desirable on why TA presented an appealing option that has no superior alternative. What is more, there is hardly a binary decision between following a TA procedure or any single alternative qualitative data analysis method. Researchers could experiment with combining elements of TA with elements of alternative qualitative data analysis. According to our results, this rarely happens or at least is rarely reported in published articles.

Another untapped potential is complementing qualitative TA with quantitatively-oriented methodologies such as content analysis (see Humble and Mozelius, 2022; Vaismoradi et al., 2013) or cluster analysis (see Rose et al., 2023). TA regularly results in countable categories, so that any TA application on data with sufficient number of units of analysis could be complemented accordingly, addressing questions such as: how often does a theme come up? How often does it come up with a positive or negative connotation? Or how often does it come up in conjunction with another theme?

What is more, the systematic element in TA does not only facilitate transparency but also communication and co-operation with researchers, who lack “qualitative research sensibility” (Braun and Clarke, 2023a, p. 701) and/or favor a more systematic, quantitative methodology. TA is thus in a prime position to facilitate multi- or mixed methods research across the quali-quanti divide, building bridges with quantitative research paradigms and epistemologies.

## Limitations of this scoping review

Three limitations of this study are particularly noteworthy. First, we only consider peer-reviewed journal articles. This excludes other relevant work, including seminal TA research published in books and book chapters. Still, we followed this document search strategy and retain this decision to ensure the overall quality of the reviewed documents. Second, the data set analyzed is composed of articles published in the top-ranked journals listed on Scopus. It is known that most international scientific databases, including Scopus, overrepresent scientific documents written in English, which are mostly coming from Western countries exhibiting a bias to the detriment of other languages and non-Western scholarship. Including articles from a larger number of communication journals including those written in Spanish, Portuguese, and other languages may have generated different results. To identify best practice, however, focusing on the most prestigious publications is particularly useful, as it provides a scope on the application and uses of TA in the most influential publications in the academic field. Third, a limited number of journals account for the bulk of articles in our data set. We cannot exclude the possibility that a small number of editors and peer communities have affected time trends, so that the generalizability of aggregate trends remains limited. These limitations should be considered when interpreting the results of our analysis.

## Conclusions and outlook

According to the current rates of growth identified in this paper, TA is at least bound to become a predominant method of manually analyzing qualitative data. This scoping review illustrates that TA does not only serve to popularize qualitative research in some principally quantitative research areas. It has also come to rapidly complement or possibly replace alternative qualitative data analysis methods in the predominantly qualitative research area of communication.

Nonetheless, our analysis also illustrates considerable challenges. There is some divergence between the TA ideal and TA practice in communications articles. A relative strength of TA in its current state is that it prescribes features of an ideal data analysis process, enabling transparency and reflexivity for specific applications. However, according to our scoping review, explicit reflexivity is not the rule even in highly cited articles. Neither do we find much apparent constructive criticism and general deliberations on how to improve TA.^19^ The increasing popularity, ease and broad scope of TA application should not lead to complacency. For instance, as TA is applied across a variety of topics and data sets, tailoring of methods to specific circumstances will be required. In the long run, continued further development is necessary for TA to sustain its momentum as a trend in qualitative data analysis. For that, TA may require a critical mass of ambitious specialists after all.

One way of evolving TA is to anchor it in the methodological literature on manual qualitative data analysis, over and beyond the seminal (Braun and Clarke, 2006). By far the most widely cited source on TA is Braun and Clarke's (2006) classic article. It is cited by over a third of all articles in our data set. No other source comes close. However, as Braun and Clarke have refined and further developed their approach, it can only be recommended to also engage with their later works and other methodological guidance. Further commonly referenced sources found in our data set are displayed in Table 5.

Another way ahead for TA is cultivating linkages with automated NLP, an area of swift development over recent years. Processes akin to TA used to be an essential aspect of the early, “familiarization” and training stages of NLP projects. TA may inform the thousands of applied and academic researchers working on NLP on how to better understand what learning algorithms do, what their limitations are, and how to improve them. As Baden et al. (2022, p. 13) note: “[W]ithout a recognition of the knowledge generated by decades of social scientific, linguistic and other text-based research, computational tool developers are likely to miss or inadequately model important textual properties, and are bound to laboriously reinvent the wheel through trial and error.” By the same token, TA researchers (and any qualitative data analyst, who continues to rely on human reading) may learn from experiences in NLP.

Also, greater transparency in terms of the handling of theory is required in TA applications. Furthermore, all indications are that TA is a versatile tool to analyze any type of qualitative data. However, the potential for analyzing various sets of complementary data on the same or closely related phenomena without having to master different data analysis methods remains largely untapped. Neither do we find attempts at replicating data analyses, which is common in quantitative research. As TA is more systematic than alternative manual data analysis methods, this may well lead to advances in qualitative research, documenting to what extent results hinge on subjective choices.

Finally, TA adopts a middle-ground between the extremes of traditional qualitative and quantitative research. It “works” as a stand-alone method, but also seems well-suited to build bridges and allow for complementary combinations. On the one hand, TA could complement less systematic methods—such as narrative analysis or discourse analysis. TA offers an accessible procedure to more transparently document aspects of data analysis, with relatively little loss of detail and nuance by “forcing” categorizations onto qualitative pieces of evidence. On the other hand, TA could also be combined with quantitative methods—such as (1) quantitative content analysis, (2) statistical analysis of the frequencies of themes subject to categorical or numerical data, for instance regarding interviewees' demographics, or even (3) NLP. On the systematic-algorithmic end, content analysis and NLP are logical TA complements for large data sets, and current AI-assisted thematic coding is a promising avenue requiring further exploration. When engaging in such analyses, TA can serve as a blueprint for preparatory steps for NLP applications, in which authors familiarize themselves with the data before running sophisticated software, or when they “train” algorithms. Combinations along these lines certainly have the potential to inspire further advances in TA.

Overall, TA proponents have established it as one of *the* workhorses of manual qualitative data analysis, distinct from quantitative content analysis and NLP, but relatively compatible with quantitative research methodology. According to our data, however, the potential for multi-method research and cross-fertilization between TA and the quickly developing field of NLP remains largely untapped. Furthermore, TA practice deviates considerably from the ostensible ideals of transparency and reflexivity, which limits its potential for evolution and adaptation. In practice, even rather successful TA users often seem to take it for granted while applying it. Breaking out of these apparent conventions will be an awarding route for some, more ambitious researchers currently considering TA.

## Data Availability

The original contributions presented in the study are included in the article/Supplementary material, further inquiries can be directed to the corresponding author.

## Footnotes

1 Fugard and Potts (2015) apply an essentially quantitative logic to discuss adequate sample sizes for what they refer to as thematic analysis. Their approach has been rejected by Hammersley (2015) and Braun and Clarke (2016) for not matching with the qualitative, exploratory and inductive methodologies associated with many TA applications.

2 In essence, ‘themes' in Braun and Clarke's TA approach are conceived as abstract entities that bring ‘meaning and identity to a recurrent experience and its variant manifestations' (DeSantis and Ugarizza, 2000, p. 362).

3 See Braun and Clarke (2013, p. 50) for a comparison of TA with other qualitative methods.

4 The journal *Cyberpsychology, Behavior, and Social Networking*, rank 35 on the SJR list, was called *CyberPsychology and Behavior* from 1998 to 2009. We included all relevant articles from this journal under both of its names.

5 There are many examples that followed this strategy (e.g. Bellibaş and Gümüş, 2019; Castillo and Hallinger, 2018; Gümüş et al., 2021).

6 The data set includes three articles from the journal *Comunicar* (SJR rank 34). These articles were published in Spanish and English.

7 We classified each of the 342 articles in our data set into ICA topical areas, based on greatest consistency with divisions' or interest groups' mission statements. Articles were classified into up to three topical areas (see Supplementary material B).

8 The theoretical-conceptual articles are authored by Pavlenko (2007), Altheide (2000), and Talmy (2011).

9 Arguably, these articles lack methodological rigor and misunderstand TA as an ‘anything goes' approach (Braun and Clarke, 2023b, p. 2).

10 Kuppens and Mast (2012) investigate a reality television programme and interviewed a producer and two audience members. As part of the large-scale EU Kids Online project, Cernikova et al. (2016) analyze the data from 114 interviewees and 56 focus groups in nine European countries.

11 We report sample sizes in greater detail for the most highly cited articles below.

12 Minimum number of citations of a cited reference: 2. Of the 16,067 cited references, 390 meet the threshold.

13 The other three publications are an empirical study about ‘the way participants use discourse to interpret their relationships' (Owen, 1984, p. 274), Goldsmith's (2004) book *Communicating social support*, and Goffman's (1959) book *The presentation of self in everyday life*.

14 In a broader ‘mapping review' of qualitative research, Bradbury-Jones et al. (2017) also document the high impact of this work.

15 Including the 2017 edition.

16 For each textbook including all prior editions.

17 We acknowledge that citation counts (aka impact) are an imperfect indicator of ‘best practice' (Leydesdorff et al., 2016).

18 Comparing the difficulty of their TA approach for a small project with interpretative phenomenological analysis, grounded theory lite, and pattern-based discourse analysis (Braun and Clarke, 2013, p. 50) rate it as “easy”.

19 A notable exception is Barkhuizen (2010), who embeds his empirical analysis in a discussion of broader research design issues (see Supplementary material D).
