# Supplementary material for: Digging in or building bridges? A scoping review of thematic analysis
Source: Front Res Metr Anal. 2025 Nov 20;10:1617380. doi: 10.3389/frma.2025.1617380 (PMC12675455; doi:10.3389/frma.2025.1617380)
Supplement: Supplementary file 1 [file Table_1.pdf]

Supplementary Material A. Journals included in the data set

Rank	Journal name	Number of articles	Article citations				Average article age[(1)]	Article citations per year since publication		
			Journal total	Mean per article	Min	Max		Mean per article	Min	Max
1	Health Communication	55	629	11.44	0	40	5.45	2.35	0.00	6.00
2	Journal of Social and Personal Relationships	27	500	18.52	0	91	7.85	2.21	0.00	15.17
3	New Media and Society	23	650	28.26	0	199	5.26	4.99	0.00	39.80
4	Information, Communication & Society	18	176	9.78	1	54	4.61	2.10	0.27	10.80
5	Public Relations Review	13	408	31.38	2	133	6.85	5.01	0.67	26.60
6	Journal of Applied Communication Research	13	255	19.62	0	47	13.69	1.34	0.00	2.20
7	Cyberpsychology, Behavior, and Social Networking[(2)]	12	509	42.42	2	319	6.75	3.61	0.50	16.79
8	Public Understanding of Science	11	483	43.91	2	121	8.00	4.93	0.50	12.86
9	Journalism Practice	11	127	11.55	0	44	3.55	2.91	0.00	11.00
10	International Journal of Communication	11	122	11.09	4	47	5.36	2.25	0.60	9.40
11	Communication Education	10	227	22.70	4	70	11.60	1.93	0.80	4.38
12	Technology, Pedagogy and Education	10	120	12.00	0	51	5.70	1.68	0.00	5.10
13	Journalism	10	66	6.60	1	15	4.10	2.16	0.14	6.00
14	Social Media + Society	9	162	18.00	4	52	5.78	2.81	0.80	7.43
15	Media, Culture & Society	9	118	13.11	2	38	5.78	3.30	0.50	12.67
16	Telematics and Informatics	7	129	18.43	2	46	7.71	2.60	0.33	6.67
17	Journal of Communication Management	7	58	8.29	1	20	7.71	1.38	0.25	5.00
18	Management Communication Quarterly	6	176	29.33	5	102	12.33	1.97	0.59	4.64
19	Journal of Professional Capital and Community	6	82	13.67	1	74	4.33	2.01	0.25	9.25
20	Journal of Health Communication	6	52	8.67	2	18	7.83	1.15	0.33	2.57
21	Journal of Family Communication	6	40	6.67	2	15	4.67	1.65	0.29	5.00
22	Convergence	6	32	5.33	3	11	3.17	1.74	0.75	3.67
23	Mass Communication and Society	4	133	33.25	2	66	8.50	3.73	0.29	8.25
24	Internet Research	4	116	29.00	3	54	4.75	6.85	1.50	18.00
25	Poetics	4	96	24.00	0	69	10.00	1.67	0.00	3.13
26	Journalism Studies	4	48	12.00	3	27	3.50	3.10	1.00	5.40
27	Crime, Media, Culture	4	23	5.75	0	19	5.25	0.74	0.00	1.46
28	Mobile Media & Communication	4	23	5.75	1	13	3.75	1.36	0.50	2.60
29	Applied Linguistics	3	665	221.67	75	459	14.67	14.14	5.36	27.00
30	Comunicar	3	33	11.00	6	18	7.33	2.06	0.82	4.50
31	Chinese Journal of Communication	3	21	7.00	2	10	7.67	0.97	0.25	1.67

32	Learned Publishing	3	12	4.00	0	6	3.33	1.33	0.00	2.00
33	Journal of Communication	2	101	50.50	11	90	23.50	2.11	0.48	3.75
34	Big Data & Society	2	43	21.50	1	42	6.50	2.46	0.25	4.67
35	Communication Monographs	2	14	7.00	5	9	4.00	2.00	1.50	2.50
36	International Journal of Press/Politics	2	14	7.00	2	12	1.50	4.00	2.00	6.00
37	Learning Environments Research	2	11	5.50	3	8	4.50	1.32	1.14	1.50
38	International Communication Gazette	2	2	1.00	0	2	5.50	0.13	0.00	0.25
39	Group Processes & Intergroup Relations	1	201	201.00	201	201	25.00	8.04	8.04	8.04
40	International Journal of Advertising	1	72	72.00	72	72	17.00	4.24	4.24	4.24
41	European Journal of Communication	1	50	50.00	50	50	24.00	2.08	2.08	2.08
42	Communication Theory	1	33	33.00	33	33	5.00	6.60	6.60	6.60
43	Journalism & Mass Communication Quarterly	1	32	32.00	32	32	17.00	1.88	1.88	1.88
44	Journal of Media Psychology	1	17	17.00	17	17	15.00	1.13	1.13	1.13
45	Communication Research	1	4	4.00	4	4	3.00	1.33	1.33	1.33
46	Digital Journalism	1	3	3.00	3	3	3.00	1.00	1.00	1.00
**Totals**		342	6,888							
**Means**[3]		7.28	146.55	26.52			7.90	2.77		

[1] 2024 minus publication year.

[2] Including Cyberpsychology and Behavior (1998–2009).

[3] The following 19 journals out of the 65 top-ranked Communication journals featured no relevant articles: Communication Methods and Measures, Political Communication, Journal of Advertising, Human Communication Research, Public Opinion Quarterly, Annals of the International Communication Association, Research on Language and Social Interaction, Media Psychology, Review of Communication Research, Vehicular Communications, Journalism & Communication Monographs, International Journal of Strategic Communication, Journal of Broadcasting & Electronic Media, Digital Communications and Networks, Journal of English as a Lingua Franca, Discourse Processes, Journal of Media Practice, Journal of Interactive Advertising, Symbolic Interaction.
