# Supplementary material for: Digging in or building bridges? A scoping review of thematic analysis
Source: Front Res Metr Anal. 2025 Nov 20;10:1617380. doi: 10.3389/frma.2025.1617380 (PMC12675455; doi:10.3389/frma.2025.1617380)
Supplement: Supplementary file 2 [file Table_2.pdf]

Supplementary Material B. Classification of TA articles according to the topical areas, based on ICA divisions and interest groups [(1)]

Count	Rank [(2)]	ICA divisions<br>(well-established topical areas with "ongoing scholarly interest")	No. of articles
1	1	Health Communication	99
2	2	Communication and Technology	93
3	3	Children, Adolescents, and the Media	41
4	4	Interpersonal Communication	39
5	5	Journalism Studies	38
6	6	Feminist Scholarship	34
7	7	Mass Communication	26
8	9	Communication Law and Policy	18
9		Organizational Communication	18
10		Political Communication	18
11	10	Ethnicity and Race in Communication	17
12	11	Intercultural Communication	11
13	12	Instructional & Developmental Communication	10
14	13	Public Relations	8
15	14	Environmental Communication	6
16		Game Studies	6
17	15	Language and Social Interaction	4
18		Popular Media & Culture	4
19	16	Global Communication and Social Change	3
20	17	Computational Methods	2
21		Philosophy, Theory and Critique	2
22	18	Mobile Communication	1
23	19	Communication History Division	0
24		Information Systems	0
25		Visual Communication Studies	0
**Count**	**Rank**	**Interest Groups**<br>(ICA topical areas with "emerging scholarly interest")	**No. of articles**
1	1	Activism, Communication, and Social Justice	11
2	2	Lesbian, Gay, Bisexual, Transgender & Queer Studies	8
3	3	Communication Science and Biology	2
4	3	Intergroup Communication	2
5	3	Public Diplomacy	2
6	4	Human-Machine Communication	1
7	4	Sport Communication	1
8	5	Media Industry Studies	0
**Aggregate**<br>(all 342 articles were classified into up to three categories)			**525**

[(1)] With more than 5,000 members in over 80 countries the International Communication Association (ICA) is the largest communication association worldwide. ICA divisions and interest groups are concerned with different aspects of media and communication scholarship and have published short ‘mission statements’ on the ICA

website. Using these mission statements as a basis to determine the topical areas which are addressed in the 342 articles under study, we allocated each article to the division(s) or interest group(s) which most closely relate to the contents of the respective article. Articles were classified as relating to either one, two or three divisions or interest groups.

(2) Ranked by number of articles.
