# Supplementary material for: Digging in or building bridges? A scoping review of thematic analysis
Source: Front Res Metr Anal. 2025 Nov 20;10:1617380. doi: 10.3389/frma.2025.1617380 (PMC12675455; doi:10.3389/frma.2025.1617380)
Supplement: Supplementary file 3 [file Table_3.pdf]

Supplementary Material C. Most cited articles from the data set: Data analysis methods

Article [1]	Topic	Citations		Data analysis methods		
		Total	Per year	TC applied	Prominent source(s)	Other methods
Pavlenko (2007)	TA compared to alternative methods for analyzing autobiographic writing in authors' second language	459	27.00	no	/	/
Coulson (2005)	Online support groups discussing shared health concerns	319	16.79	yes	Boyatzis (1998)	/
Drury & Reicher (1999)	Anti-poll tax demonstrations	201	8.04	yes	Kellehear (1993)	Consensual account construction (Denzin, 1989)
Cotter (2019)	Online communications among 'Instagram influencers' on how 'rules encoded in [recommendation] algorithms' affect visibility	199	39.80	yes	/	/
Xiong et al. (2019)	'Message frames' of social movement organizations; the case of #MeToo movement	133	26.60	yes	Braun & Clarke (2006)	Semantic network analysis and 'correlation tests' (statistics)
Talmy (2011)	Conceptualizations of 'interviews as research instruments' versus 'interviews as social practice'; data are drawn from an ethnography of a Hawai'i high school	131	10.08	yes	e.g.: Pavlenko (2007); Boyatzis (1998); Braun & Clarke (2006)	/
Smith & Joffe (2013)	Attitudes to global warming	121	11.00	yes	Joffe (2011)	/
Jaspal & Nerlich (2014)	UK press coverage of 'socio-political dimensions of fracking'	111	11.10	yes	/	/
Ruckenstein & Pantzar (2017)	Quantified Self	107	15.29	yes	/	/
Kassing (2002)	'[U]pward dissent within (…) organizations'	102	4.64	yes	Glaser & Strauss (1967)	/
Antony & Thomas (2010)	Online communications regarding a police shooting in the USA	100	7.14	yes	Orbe & Kinefuchi (2008)	/
Hinchliff & Gott (2004)	Sexual health; 'sexual activity in the context of long-term marriage'	91	4.55	yes	Ritchie & Spencer (1994)	/
LeFebvre (2018)	Relationship initiation on Tinder	91	15.17	yes	E.g.: Bulmer (1979); Corbin & Strauss (1990)	Descriptive statistics
Harwood & Lin (2000)	Grandparents' relationships with their grandchildren	90	3.75	yes	/	/
Veltri & Atanasova (2017)	Attitudes regarding climate change	90	12.86	no[2]	/	/
Coulson & Knibb (2007)	Participation in an online support group regarding food allergies	86	5.06	yes	Boyatzis (1998)	/
Barkhuizen (2010)	'The imagined "better life" of a migrant pre-service teacher'	75	5.36	yes	Pavlenko (2007)	/
Rincón-Gallardo & Fullan (2016)	Education networks	74	9.25	yes	Boyatzis (1998)	/
Roberts & Pettigrew (2007)	Childhood obesity and advertising	72	4.24	yes	/	Content analysis
Morreale & Pearson (2008)	'Communication instruction' and 'students' future personal and professional success'	70	4.38	yes	/	/
Tandon et al. (2021)	Fear of missing out among social media users	54	18.00	no	/	Content analysis of prior studies that adopted TC
Peña Gangadharan & Niklas (2019)	'Norms, values, and practices among European civil society organizations' regarding 'data and discrimination'	54	10.80	yes[3]	Boyatzis (2001)	/

Suzor et al. (2019)	Transparency in content moderation on online platforms	47	9.40	yes	/	Content analysis
Jamil (2020)	'Pakistani female journalists' lived experiences of sexual harassment, threats and discrimination'	44	11.00	yes	/	/
Ytre-Arne & Moe (2021)	'Perceptions of algorithms on the media'	38	12.67	yes	DeVito et al. (2017)	/
Gaudette et al. (2021)	Extreme right activists on Reddit	36	12.00	yes	Braun & Clarke (2006)	/
Jones et al. (2020)	'Ideology and rhetoric of [… a] Manosphere group' and how it 'propagates and normalises misogynistic beliefs'	34	8.50	yes	/	Content analysis
Lupinacci (2021)	Experiences with social media regarding 'continuous connectedness'	25	8.33	yes	Attride-Stirling (2001); Guest et al. (2012)	/
Timmermans et al. (2021)	'Mobile daters' […] experiences with ghosting'	25	8.33	yes	Braun & Clarke (2006)	Content analysis and inferential statistics

(1) The table covers all top-20 most cited papers and all top-20 papers by citations per year

(2) Automatic thematic analysis (text mining), combined with semantic network analysis, and 'text classification according to psychological process categories'

(3) '[N]etworked thematic analysis' with an 'iterative strategy'
