# Supplementary material for: Digging in or building bridges? A scoping review of thematic analysis
Source: Front Res Metr Anal. 2025 Nov 20;10:1617380. doi: 10.3389/frma.2025.1617380 (PMC12675455; doi:10.3389/frma.2025.1617380)
Supplement: Supplementary file 4 [file Table_4.pdf]

Supplementary Material D. Most cited articles from the data set: Theory and data collection

Article[(1)]	Theory		Data	
	**Use of theory**	**Specifics (examples)**	**Collection methods**	**Count of units of analysis**
Pavlenko (2007)	n.a. (paper is on data analysis methods)	/	n.a.	n.a. *(not a systematic literature review)*
Coulson (2005)	'[D]eductive (i.e., theory-driven) thematic analysis'	Categories of social support (Cutrona & Suhr 1992)	Web scraping	'572 messages posted to a computer-mediated bulletin board for individuals affected by irritable bowel syndrome'
Drury & Reicher (1999)	Deductive	Social identity model of crowds (e.g. Reicher 1982)	Interviews	Interviews with 29 individuals, partially in groups, 'video footage', and 'newspaper accounts'
Cotter (2019)	Mostly inductive [(2)]	/	Web scraping	Not specified
Xiong et al. (2019)	Deductive	Co-creation (e.g. De Moya & Bravo 2016), feminism (e.g. Aldoory 2005), and framing (Hon 2016)	Web scraping	21 Twitter accounts, featuring 408 relevant tweets
Talmy (2011)	Focus is directed on the conceptualization of the interview as research instrument	/	Interviews	Three groups of ESL students (in total 30 interviews)
Smith & Joffe (2013)	'[I]nductive' and 'deductive' coding	Social representations theory (Markova 2003)	Interviews	'Purposive sample' of 56 interviewees, with the help of a 'London-based recruitment agency'
Jaspal & Nerlich (2014)	Deductive	Social representations theory (Moscivici 1963)	Document analysis	341 articles from 4 British broadsheet newspapers
Ruckenstein & Pantzar (2017)	Deductive	Kuhn's (1962) paradigms	Document analysis	41 *Wired* articles
Kassing (2002)	Deductive, even though the author refers to a 'grounded theory methodology'	Exit-voice-loyalty model of employee dissatisfaction (Hirschman 1970)	Survey	135 employees of an undisclosed number of organizations in Arizona
Antony & Thomas (2010)	Deductive	[G]uard dog versus 'watchdog' conceptualizations of the role of mainstream media (e.g. Donohue et al. 1995)	Web scraping	4 cellphone videos; 82 comments on YouTube
Hinchliff & Gott (2004)	Inductive	Reporting of prior empirical results and drawing on a framework provided by the National Centre for Social Research, UK	Interviews	'28 participants, who have been married for a minimum of 20 years' from Sheffield, UK
LeFebvre (2018)	Self-declared 'exploratory' but with some deductive use of theory	Relationship development model (Knapp & Vangelisti, 2010)	Survey	395 participants
Harwood & Lin (2000)	Inductive (reporting of prior empirical results)	/	Survey	131 grandparents of young adults enrolled at a U.S. university
Veltri & Atanasova (2017)	Deductive	'[P]ersonal public' (Schmidt 2014); 'social representation theory' (Moscivici 2000)	Web scraping	Over 61,122 tweets of a 'random week sample'
Coulson & Knibb (2007)	Inductive; explictly explorative	/	Interviews (online, structured)	32 members of the Food Allergy Survivors Together online support group
Barkhuizen (2010)	Focus is directed on positioning analysis (of narrative data)	/	Reflective narratives, narrative interviews, conversations	A single migrant (from Tonga to New Zealand), aspiring to become an English teacher
Rincón-Gallardo & Fullan (2016)	Mostly inductive; described as an	'[S]ocial physics' and 'reality mining' (Pentland 2014);	Literature review	6 generic 'literature reviews/studies' and academic reports on 12 'network case studies' regarding education

	‘iterative process of thematic analysis’			
Roberts & Pettigrew (2007)	Inductive	/	Broadcasting media analysis	212 food advertisements ‘screened in Perth, Australia, during children's morning television programming’
Morreale & Pearson (2008)	Limited deduction; referring to extant ‘themes’ in the literature	/	Academic and non-academic literature review	93 ‘journal and newspaper articles, reports and surveys’
Tandon et al. (2021)	Some deduction by comparing and recombining extant themes	/	Systematic literature review	58 empirical studies obtained from four academic databases
Peña Gangadharan & Niklas (2019)	Limited deduction	Abnormal justice (Fraser 2010)	Interviews	30 interviews with civil society representatives
Suzor et al. (2019)	Inductive	The ‘Santa Clara Principles’ (2018) provide an initial structure	Survey	380 respondents, ‘who have been the subject to content moderation practices of digital platforms’
Jamil (2020)	Deductive	Postcolonial feminist theory (Mohanty 2003) and intersectionality	‘In-depth interviews’ and focus groups; detailed discussion of inter-coder reliability	41 female journalists; purposive sampling
Ytre-Arne & Moe (2021)	Limited deduction; ‘[p]redominantly inductive’	‘[F]olk theories’ (Eslami et al. 2016), ‘algorithmic imaginaries’ (Bucher 2017)	Survey (data comes from a survey regarding media literacy)	1,363 Norwegians; representative sample
Gaudette et al. (2021)	Mostly inductive	‘[S]ocial movement theory’ (e.g. Futrell and Simi 2004) and ‘social constructionist paradigm’ (Snow 2001)	Web scraping	1,000 ‘highly upvoted user-submitted comments, and 1,000 random user-submitted comments’
Jones et al. (2020)	Deductive	Toxic masculinity (Haider 2016) and ‘hegemonic masculinity’ (Connell and Messerschmidt 2005)	Web scraping	1,550 tweets from 3 very active ‘users on Twitter’, 138 responding tweets, and a specific website (extensive discussion of intercoder reliability)
Lupinacci (2021)	Inductive; referring to prior empirical work	/	Interviews (diary interviews)	5-day diary interviews of 20 participants living in London; explicitly not representative
Timmermans et al. (2021)	Inductive; referring to prior empirical work	/	Survey; open and closed questions	328 mobile daters

(1) The table covers all top-20 most cited papers and all top-20 papers by citations per year

(2) Moving beyond simple notions of influencers ‘gaming the system’ to ‘playing the visibility game’, where the latter allows for greater agency of other parties than the influencers and their opportunistic responses to known algorithmic processes
